# The effect of the platelet-rich plasma and ozone therapy on tendon-to-bone healing in the rabbit rotator cuff repair model

**DOI:** 10.1186/s13018-021-02320-w

**Published:** 2021-03-19

**Authors:** Murat Gurger, Gokhan Once, Erhan Yilmaz, Sukru Demir, Ilknur Calik, Yakup Say, Ahmet Kavakli, Sefa Key, Mustafa Umit Gurbuz, Onur Bingollu

**Affiliations:** 1 Department of Orthopaedics and Traumatology, Faculty of Medicine, Firat University, 23190 Elazig, Turkey; 2 Department of Medical Pathology, Faculty of Medicine, Firat University, 23190 Elazig, Turkey; 3 Department of Metallurgical and Materials Engineering, Tunceli University, 62000 Tunceli, Turkey; 4 Department of Anatomy, Faculty of Medicine, Firat University, 23190 Elazig, Turkey

**Keywords:** Platelet-rich plasma, Ozone therapy, Tendon-to-bone healing, Rotator cuff

## Abstract

**Background:**

The aim of this study is to histologically and biomechanically investigate the effects of local PRP and ozone therapy (O_2_O_3_) on tendon-to-bone healing in a rabbit model of the supraspinatus tendon tear.

**Methods:**

Four groups were formed to have seven rabbits in each group: repair, R; repair + PRP, RP; repair + ozone, RO; and repair + PRP + ozone, RPO. The supraspinatus tendon was detached by sharp dissection from the footprint and an acute tear pattern was created. Thereafter, tendon repair was performed with the transosseous technique. In the RP group, PRP, and in the RPO group, PRP + O2O3 mixture was injected to the tendon repair site. In the RO group, O_2_O_3_ gas mixture was injected into subacromial space three times a week for a total of 4 weeks. The study was ended at postoperative 6th week.

**Results:**

When compared with the R group, a statistically significant increase was observed in the biomechanical strength of the RP and RPO groups. The highest increase in biomechanical strength was detected in the RPO group. The histology of the RO and RPO groups showed better collagen fiber continuity and orientation than the R and RP groups.

**Conclusions:**

The results obtained from this study show that the ozonized PRP can be used as biological support to increase tendon-to-bone healing. However, these results need to be supported by clinical studies.

## Introduction

Rotator cuff tears are among the most common causes of shoulder pain and function loss in adults. Although there is considerable improvement in surgical techniques, significant healing problems are observed after surgical repair [1]. In addition, the structure, composition, and organization of rotator cuff insertions cannot be fully reconstructed after surgical treatment [1]. The tendon heals with an intermediate layer containing fibrovascular scar tissue in the region where it adheres to the bone [1]. If the healing is successfully completed, this intermediate layer is gradually transformed to the Sharpey-like collagen fibers that bind the tendon to the bone [1].

In recent years, there has been an increasing interest in biological augmentations used for tendon healing. Perhaps the most interesting of these is platelet-rich plasma (PRP) [2–7]. In some in vitro studies, PRP has been shown to increase mesenchymal stem cells [8]. Therefore, the use of PRP has become attractive to prevent healing problems, especially after rotator cuff repair. Similarly, ozone therapy (O_2_O_3_) is also an effective method for musculoskeletal diseases. Ozone is a very reactive gas that is toxic to the respiratory system, but it can be dissolved in the blood at appropriate doses and triggers important molecular, biochemical, and pharmacological pathways. Therefore, it is used in the treatment of many diseases such as age-related macular degenerations, ischemic and infectious diseases, intervertebral disc herniations, and wound healing problems [9, 10]. In addition, ozone therapy alone has been shown to improve acute or chronic tendinitis even in the presence of calcium deposits [11]. Ozone stimulates many biochemical mechanisms generated by tendon cells and endothelial cells. It also provides absorption of calcium deposits and destroys arachidonic acid, creating a massive anti-inflammatory effect [11]. Although there are studies showing that ozone therapy is used in the treatment of acute and chronic tendonitis, to our knowledge, no prior studies have examined the use of ozone therapy after supraspinatus tendon repair.

The aim of this study was to determine the histological and biomechanical effects of local PRP and ozone therapy on tendon-to-bone healing in rabbit model of the supraspinatus tendon tear.

## Materials and methods

After receiving an approval form from the local ethics committee for animal experiments, 4-month-old 32 New Zealand type rabbits weighing 3–3.5 kg were randomly divided into 4 equal groups with 7 animals in each group. In order to obtain reference values, the right shoulders of 4 healthy rabbits were used for histological studies and the left shoulders were used for biomechanical studies. Groups are set as specified in table 1. It was confirmed by the literature that the application of the same surgical procedure on both shoulders of rabbits did not severely restrict the feeding and movement of animals [12, 13]. Then, rotator cuff repair was performed on both shoulders of all rabbits except four. The rabbits were kept in individual cages in the institution’s animal experiments research center and given ad libitum access to water and standard diet.

**Table 1:** Experiment groups

Groups	Definition
R (*n* = 7)	Rotator cuff repair is performed, no additional treatment is given
RP (*n* = 7)	Rotator cuff repair is performed, PRP treatment is given
RO (*n* = 7)	Rotator cuff repair is performed, ozone treatment is given
RPO (*n* = 7)	Rotator cuff repair is performed, PRP + ozone treatment is given

### PRP preparation

PRP preparation was performed simultaneously during surgical procedures. All animals were given general anesthesia using a combination of ketamine (15 mg/kg, IM) and xylazine hydrochloride (5 mg/kg, IM). In the RP and repair + PRP + ozone (RPO) groups, 5 ml of blood was drawn from the marginal vein of the ear and mixed with 1 ml of sodium citrate (3.8%). PRP was prepared using the method described in the literature previously [14, 15]. Briefly, the tube containing whole blood and citrate was first centrifuged at 1600 rpm for 10 min. The first centrifugation resulted in a red and opaque layer at the bottom of the tube. This layer consists of red blood cells, white blood cells, and platelets and is called blood cell component (BCC). At the top of the tube, a yellow fuzzy layer was formed. This layer is composed of plasma and platelets and is called a serum component (SEC). The whole SEC layer and the upper 6–8 mm portion of the BCC layer were drawn into a sterile citrate-free vacuum tube. This tube was centrifuged again for 10 min at 2000 rpm. As a result of the second centrifugation, platelet poor plasma (PPP) formed in the upper 5.5 ml of the tube was aspirated. The remaining 0.5 ml was used as PRP.

### Ozone therapy

Ozone was generated using an ozone generator (Humazona, Humares GmbH Bruchsal/Germany) where a spectrometer was placed to allow for real-time control of the flow rate and concentration. A mixture of 0.5 ml of O_2_O_3_ (30 μg/mL O3 concentration) was injected into the subacromial space under general anesthesia for a total of 4 weeks three times a week, the first dose being given the 1st day after surgery [16–18].

### PRP + ozone therapy

The 0.5 mL O_2_O_3_ gas mixture (30 μg/mL O_3_ concentration) was collected with a silicone-coated disposable syringe and immediately withdrawn to a second injector containing 0.5 ml PRP via “y connector”. This mixture was injected intraoperatively to the supraspinatus repair site in the RPO group [17, 18]. 0.5 ml PRP + 0.5 ml O_2_O_3_ mixture were injected into the supraspinatus repair area in RPO group, in a single dose immediately after supraspinatus repair.

### Surgical technique

**Fig. 1:**
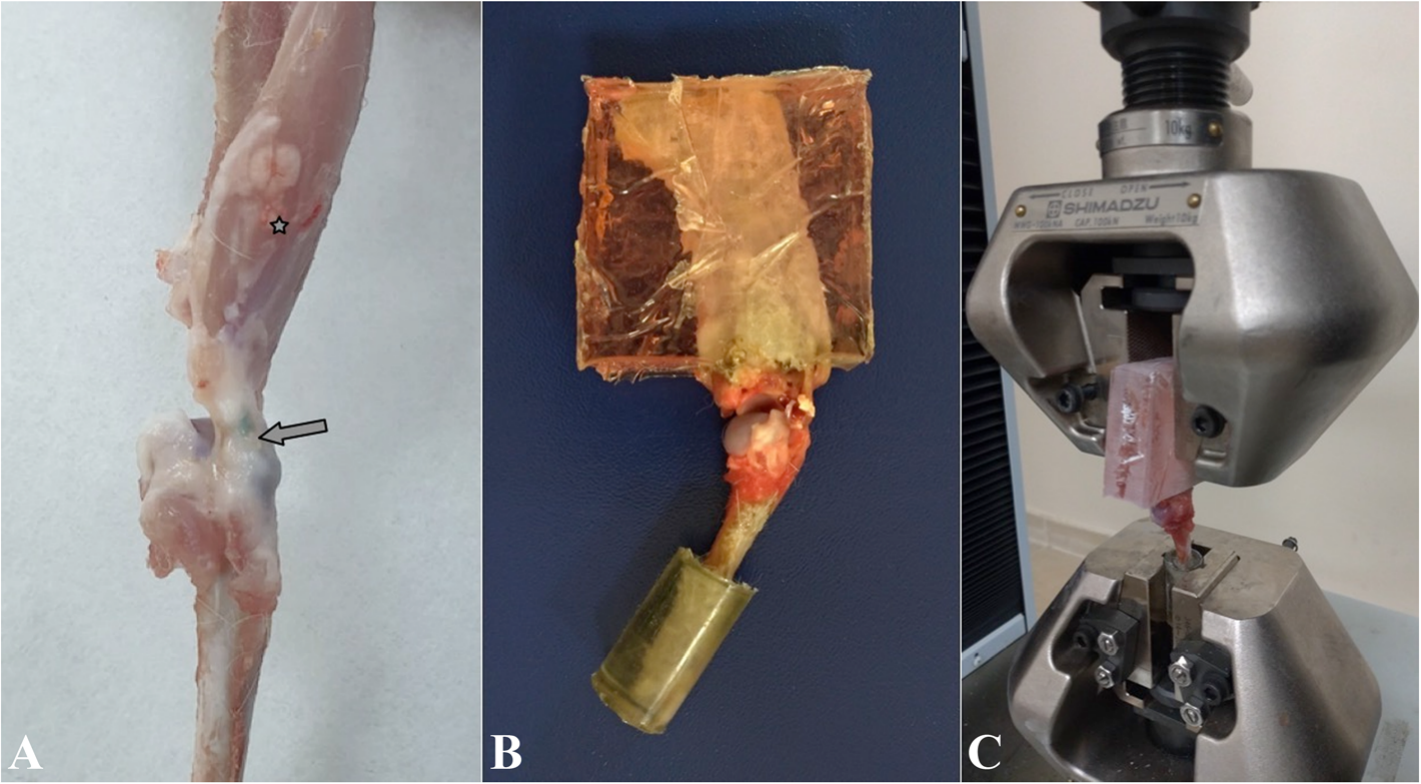
**a** Supraspinatus tendon was isolated and sutured and then detached from the footprint. **b** Bone tunnels were created. **c** Suture tips were passed through bone tunnels. **d** Suture tips were tied over the lateral humeral cortex

All animals undergoing surgery were anesthetized as described above. Sixty milligrams per kilogram IM ceftriaxone was administered to rabbits for surgical antimicrobial prophylaxis. The rabbits were taken in the lateral decubitus position and the surgery was performed under sterile conditions. The shoulder was opened with a deltoid split incision. Supraspinatus tendon was isolated and fixation suture was applied using 3-0 Polydioxanone (Ethicon, Johnson and Johnson). Then, the tendon was carefully detached from the greater tuberosity by sharp dissection. Bone tunnels were created at the anterior and posterior edges of the footprint and 5 mm lateral from the articular surface. The suture tips were tied over the lateral humeral cortex after passing through the bone tunnels. Thus, anatomic repair of the supraspinatus tendon was accomplished (Fig. 1). Following the anatomic repair, 0.5 ml PRP and PRP + O_2_O_3_ mixture were injected into the supraspinatus repair area in the RP group and RPO group, respectively. No additional treatment was applied to repair (R) and repair + ozone (RO) groups during repair. The deltoid and skin were sutured using interrupted 3-0 absorbable suture. Buprenorphine (0.05 mg/kg) was administered subcutaneously for postoperative analgesia. Feeding, weight-bearing, and intra-cage activities of the animals were not restricted.

### Tissue preparation and evaluation

At the end of the study period, 6 weeks postoperatively, the animals were sacrificed with a 10% potassium chloride intra-cardiac injection under general anesthesia. The supraspinatus tendon was carefully dissected from the surrounding tissues by preserving the scapular origo and humeral insertion. The right shoulders of the animals were used for histopathological examinations and the left shoulders for biomechanical examinations.

### Histopathological evaluation

Surgical materials were fixed in 10% neutral-buffered formaldehyde for 24 h at room temperature and then decalcified with EDTA-hydrocloric acid for 48 h. After decalcification, tissues were embedded in paraffin in the coronal plane after routine follow-up. Five-micrometer-thick serial sections were cut using a microtome. All sections were stained with hematoxylin-eosin (H&E) and MTC (Roche Ventana Trichrom staining kit). The cross-sections were examined under a microscope (BX51, Olympus, Tokyo, Japan). Digital images were taken using Olympus DP Controller system (Olympus Corporation). The measurements were performed by one pathologist who was blind to the experimental groups. The pathologist assessed the vascularity, cellularity, collagen fiber continuity, and proportion of fibers oriented parallel to the tendon-to-bone interface based on the methods described by Chung et al. [19]. Also, the rate of inflammation was evaluated. The histological findings were graded semi-quantitatively into 4 degrees (grades 0, 1, 2, and 3). The collagen fiber continuity and collagen fibers oriented parallel were graded as present with < 25% of proportion (grade 0), 25–50% of proportion (grade 1), 50–75% of proportion (grade 2), and > 75% of proportion (grade 3). Vascularity, cellularity, and inflammation rate were graded as absent or minimally present (grade 0), mildly present (grade 1), moderately present (grade 2), and severe or markedly present (grade 3).

### Biomechanical analysis

**Fig. 2:**
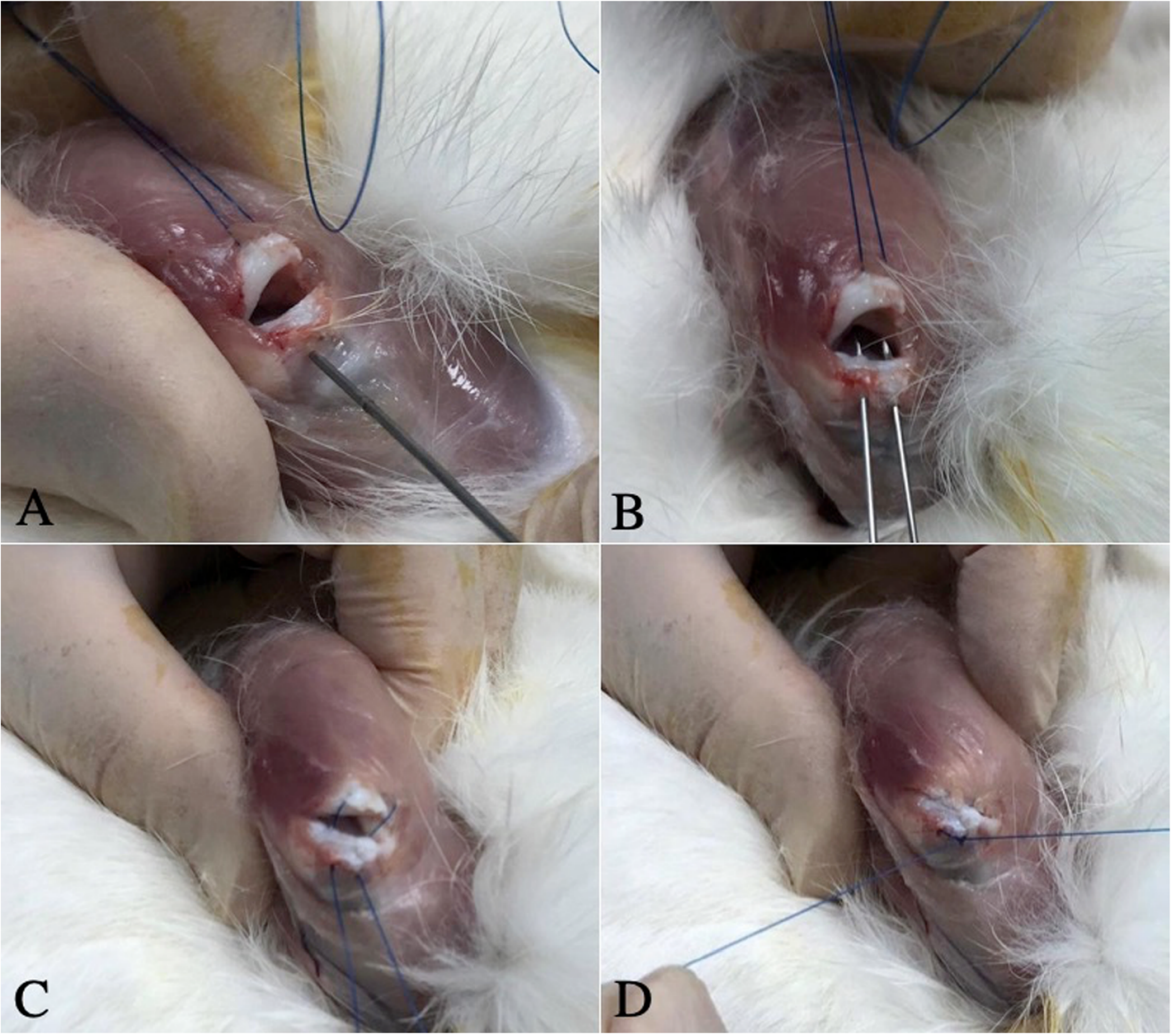
**a** A sample of the repaired supraspinatus tendon (asterisk, scapular origo; arrow, humeral insertion). **b** Polyester molded sample. **c** Tensile test

Tensile test was applied to each of the samples in the groups and the force-elongation values of the tendons were obtained. Biomechanical tests were executed by Shimadzu AG-X universal test machine with the help of 10-kN load cell and experiments were undertaken at a rate of 5 mm/min. Samples were maintained at − 80 °C until mechanical tests were performed. On the day of the tests, the tendons were allowed to dissolve at room temperature and intermittently soaked with Ringer’s solution to prevent them from drying out. Before the test, the samples were taken into a polyester mold to prevent the specimens from sliding off the heads (Fig. 2).

### Statistical analysis

After data collection, statistical analyses were performed by using SPSS 21.0 package program. Kolmogorov-Smirnov and Shapiro-Wilk normality tests were used to determine the distribution of continuous variables. Kruskal-Wallis test and *H* test with post hoc test were used to compare more than two independent groups that did not comply with normal distribution, while Man-Whitney *U* test was used to determine the relationship between two independent groups. Numerical data were expressed as mean ± standard deviation and median (minimum–maximum), qualitative data as percentages. *p* < 0.05 was considered significant.

## Results

### Histopathologic results

**Fig. 3:**
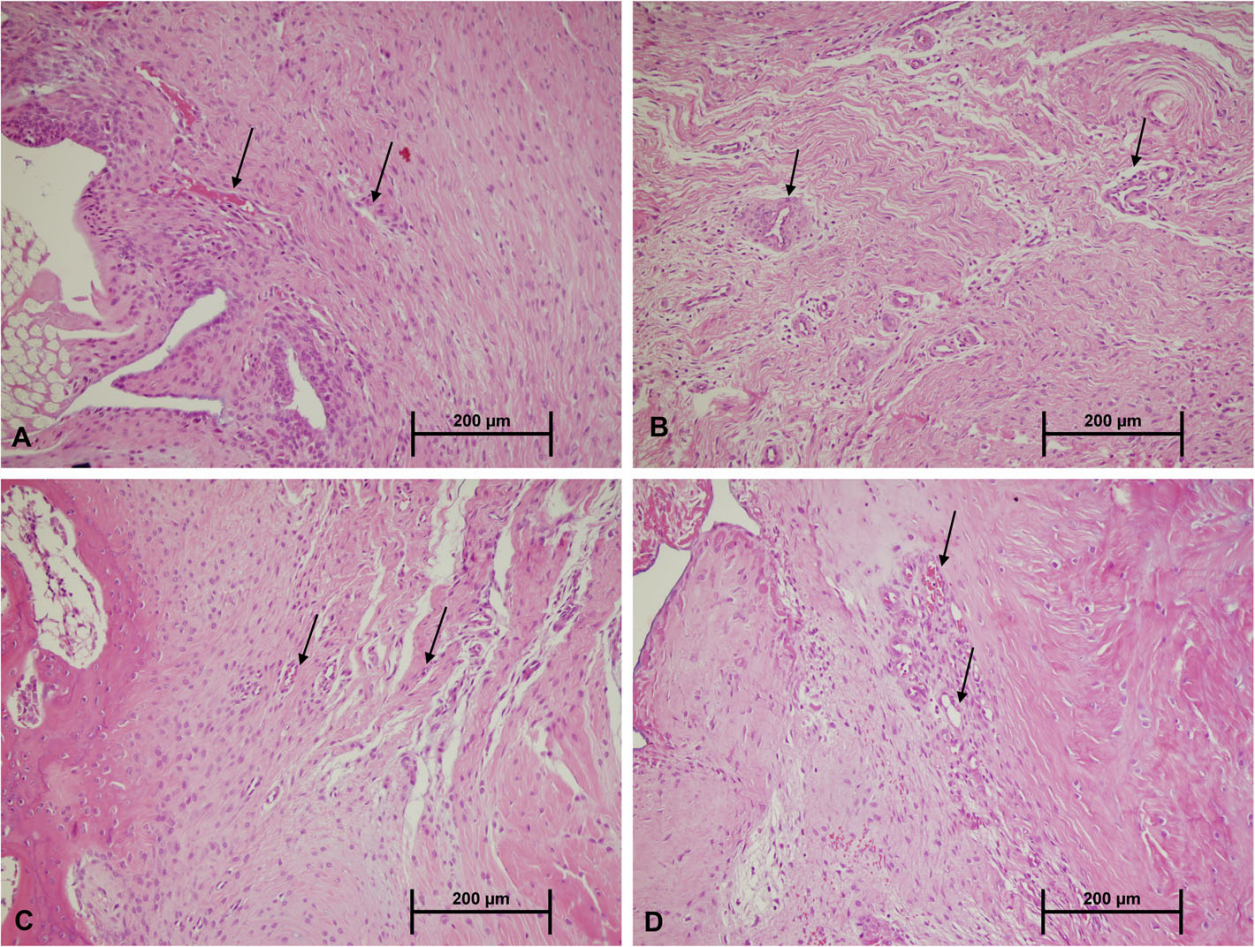
Tendon-to-bone interface H&E staining shows that vascularity and cellularity were higher with granulation tissue formation in the ozone-treated groups (**c**, **d**) compared to the non-treated groups (**a**, **b**). The arrows indicate the vascular structure

**Fig. 4:**
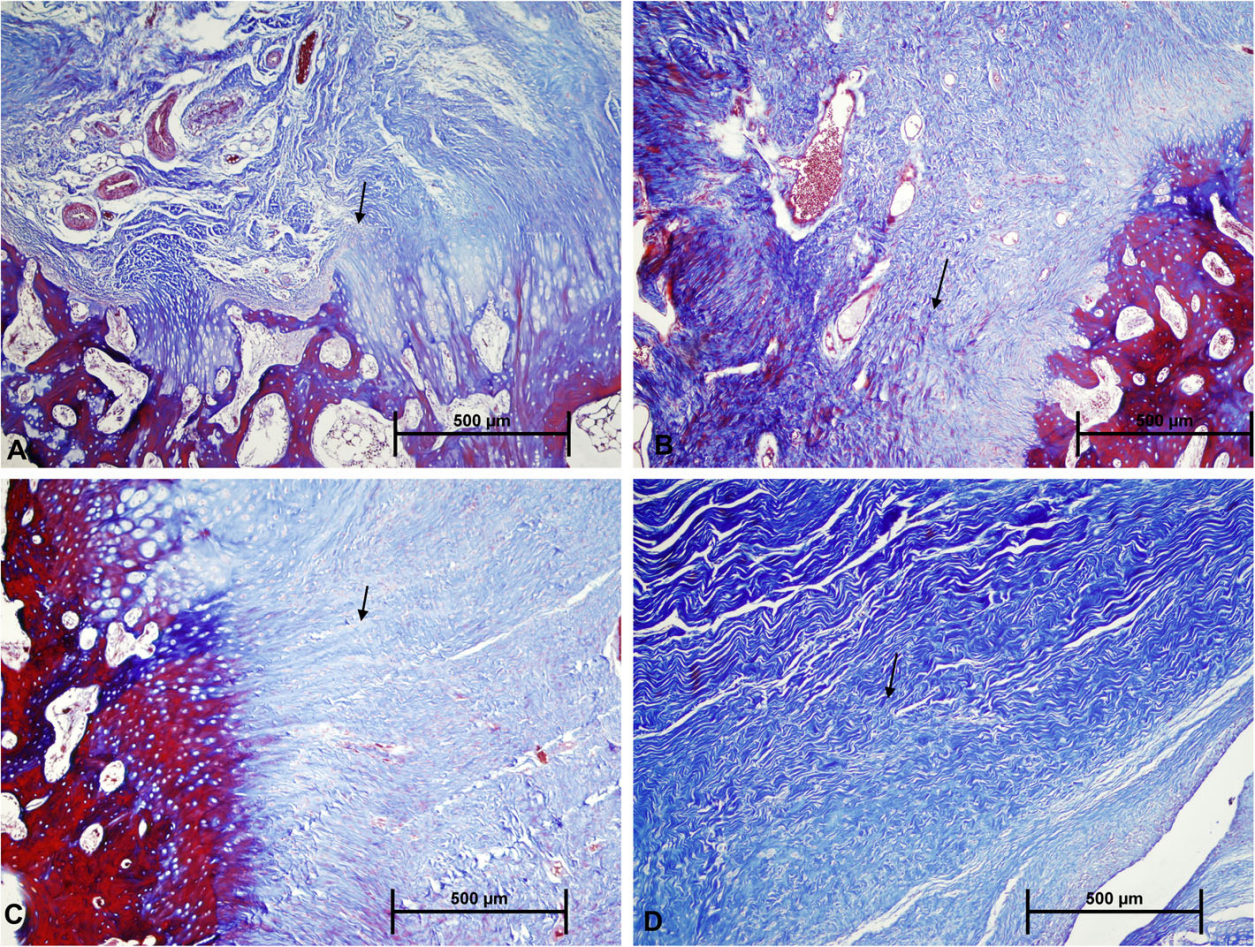
MTC staining demonstrates collagen fiber continuity and arrangement at tendon-to-bone interface. There is an improved collagen fiber continuity and arrangement in the ozone-treated groups (**c**, **d**) compared to the non-treated groups (**a**, **b**) (arrows, collagen fibers)

The results of histological grading are described in table 2. Cellularity and vascularity were found to increase in different degrees in all the groups compared to the R group. However, the increase was more pronounced in the groups treated with ozone (RO and RPO) (Fig. 3). Inflammation rate was higher in the R and RP groups compared to other two groups. Collagenization was evaluated by MTC staining (Fig. 4). The healthy tendon showed intense regular collagen fibers with full continuity to the bone. In all groups except the ozone-treated groups (RO and RPO), the collagen fibers were organized poorly, and fiber continuity was not established. In the RO and RPO groups, the number of collagen fibers was greatly increased, and there was a visible improvement in collagen organization. Also, collagen fibers that bridged the interface were visible in all 4 groups. However, the ozone-treated groups showed better collagen fiber continuity. While none of the groups could fully reach the level of the healthy rabbits in terms of the regularity of the newly formed collagen fibers and continuity to the bone, the RPO group showed the closest similarity. The collagen fibers in the interface were more perpendicular from the bone in the ozone-treated groups (RO and RPO).

**Table 2:** Results of histopathological evaluation

Parameters	R group (*n* = 7)				RP group (*n* = 7)				RO group (*n* = 7)				RPO group (*n* = 7)			
	G0	G1	G2	G3	G0	G1	G2	G3	G0	G1	G2	G3	G0	G1	G2	G3
Vascularity	1	3	2	1	0	3	3	1	0	1	3	3	0	0	5	2
Cellularity	0	5	2	0	1	3	3	0	0	4	2	1	0	2	3	2
Collagen fiber parallel arrangement	4	3	0	0	4	3	0	0	0	4	2	1	0	3	3	1
Collagen fiber continuity	3	3	1	0	2	4	1	0	2	3	1	1	2	2	2	1
Inflammation	5	2	0	0	4	2	1	0	5	2	0	0	6	1	0	0

*R* repair, *RP* repair + PRP, *RO* repair + ozone, *RPO* repair + PRP + ozone-treated group, *G0* absent-minimal, *G1* mild, *G2* moderate, *G3* marked degree

### Biomechanical results

**Fig. 5:**
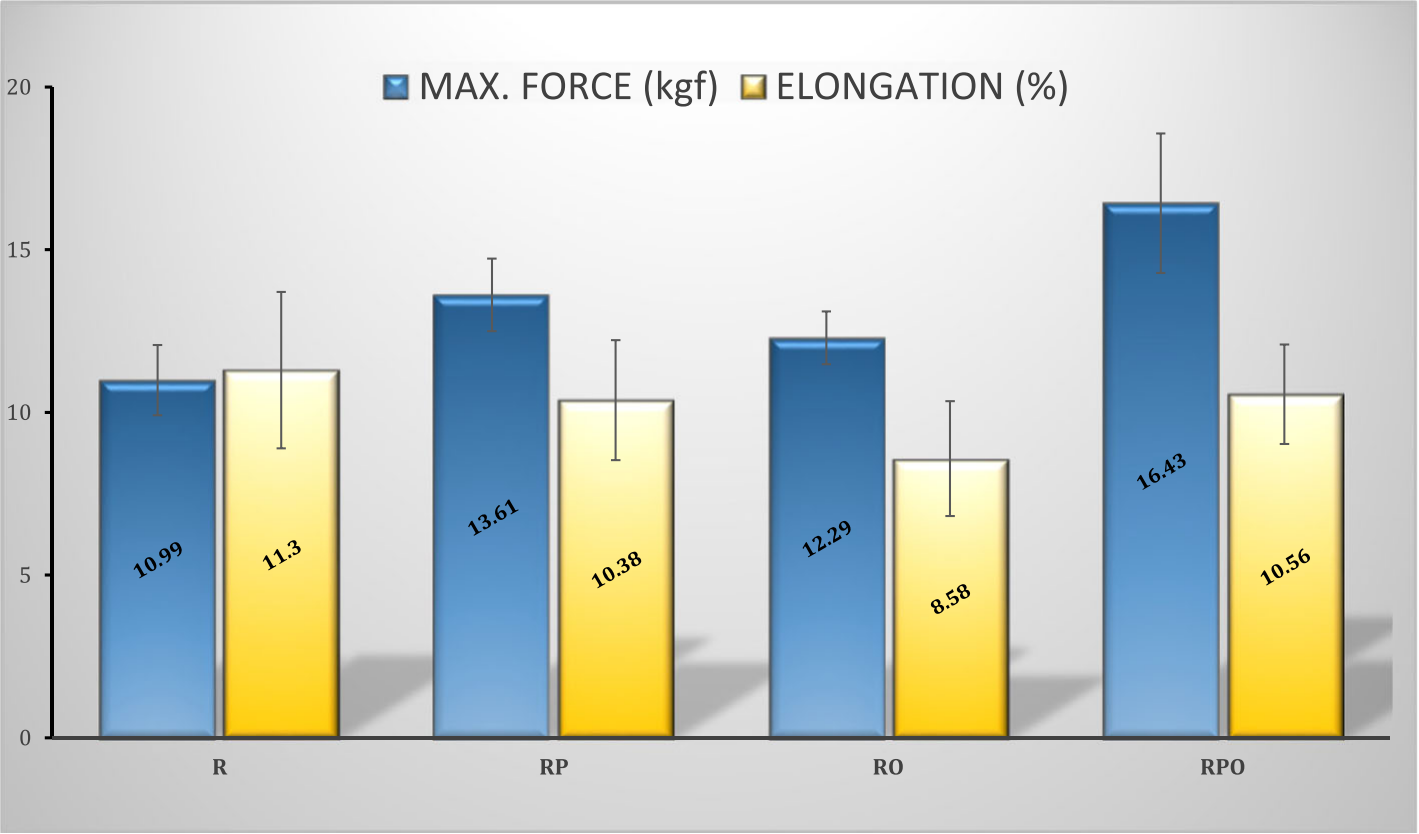
Maximum force to failure and maximum elongation (%) values of sample groups (mean ± standard deviation)

In Fig. 5, maximum force to failure (kgf) and maximum elongation (%) values are given. In samples obtained from healthy rabbits, an undamaged supraspinatus had an average tensile strength of 21.35 kgf and an elongation of 8.19% before rupture. Compared to the intact supraspinatus tendon, the strength of the R group was almost halved (10.99 kgf). When compared with the R group, tensile strength of the groups except the RO group showed a statistically significant increase. Along with this, the highest increase in tensile strength was detected in the RPO group. The statistical significance of the results obtained is summarized in table 3. When the maximum elongation values were analyzed, no statistically significant difference was found between the groups (Table 4).

**Table 3:** Results of Bonferroni post hoc analysis for maximum force to failure for each group

	R group	RP group	RO group	RPO group
R group		0.014a	0.172	0.001a
RO group	0.172	0.564		0.016a
RP group	0.014a		0.564	0.182
RPO group	0.001a	0.182	0.016a	

*R* repair, *RP* repair + PRP, *RO* repair + ozone, *RPO* repair + PRP + ozone; statistically significant: *P* < 0.05

**Table 4:** Results of Bonferroni post hoc analysis for maximum elongation (%) for each group

	R group	RP group	RO group	RPO group
R group		0.398	0.052	0.495
RO group	0.052	0.384		0.264
RP group	0.398		0.384	0.871
RPO group	0.495	0.871	0.264	

*R* repair, *RP* repair + PRP, *RO* repair + ozone, *RPO* repair + PRP + ozone; statistically significant: *P* < 0.05

## Discussion

The aim of this study was to investigate the effects of local PRP and ozone treatment on tendon-to-bone healing in rabbit model of the supraspinatus tendon tear. Our histological and biomechanical results showed that tendon-to-bone healing was more prominent in the RPO group.

Platelet-rich plasma is an autologous platelet concentrate containing various growth factors that stimulate tendon healing [20]. The number of platelets in PRP is approximately 3–5 times higher than in blood [21]. PRP contains many growth factors that are critical to tendon-to-bone healing such as transforming growth factor-β (TGF-β), basic fibroblast growth factor (bFGF), platelet-derived growth factor (PDGF), vascular endothelial growth factor (VEGF), connective tissue growth factor (CTGF), and epidermal growth factor (EGF). In addition, in vitro studies have demonstrated the ability of PRP to increase the local concentration of mesenchymal stem cells, macrophages, and fibroblasts that contribute to the healing process. Fibrin, fibronectin, and vitronectin in PRP are known as cell adhesion molecules and play important roles in connective tissue matrices [6]. Most studies investigating the effect of PRP on tendon healing and using animal models suggest that PRP is effective on tendon healing and tendon-to-bone healing [19, 22–24]. Our study was consistent with the literature, we observed that PRP increased cellularity and vascularity, an indicator of tissue healing at the repair site, and also improved biomechanical strength of the repaired supraspinatus tendon. These positive effects obtained in experimental studies are not always observed in clinical studies; thus, there is no consensus on the efficacy of PRP in clinical practice [6, 25–29]. Snow et al. found that PRP application after rotator cuff repair did not improve the functional results of the patients and the constant scores after 1 year [30]. In their meta-analysis, Zhao et al. stated that leukocyte-poor PRP significantly reduced postoperative retear rate in the mid- and long-term regardless of tear size and repair method but did not have a significant effect on postoperative pain and functional results [31]. Chen et al., in their meta-analysis, stated that patients who underwent PRP had low long-term rotator cuff retear rates had significant increase in shoulder scores, which could positively affect clinical results, but it was difficult to make a definite judgment due to the heterogenicity and poor methodological quality of the studies [32].

Ozone (O_3_) has excellent oxidizing activity as a soluble gas. In contact with biological fluids, it provides the formation of lipid oxidation products as well as reactive oxygen species [33]. Both of these products interact with white blood cells to initiate the formation of proteins, cytokines, and red blood cells. Ozone therapy (O_2_O_3_) stimulates neoangiogenesis and increases the oxygenation of tissues. This allows the tissue to heal and renew itself. Because of these properties, ozone therapy is used in the treatment of many musculoskeletal diseases including muscles, tendons, and joints. It can be injected via periarticular, intraarticular, or percutaneous [33, 34]. In the literature, ozone therapy has been used in many shoulder pathologies such as subacromial bursitis, calcified tendinitis, adhesive capsulitis, and partial rotator cuff tears, with positive results reported [33, 35]. In our study, only ozone-treated group (RO group) showed a significant increase in collagen fibers and their organization compared to the repair group (R), but when biomechanical strengths were assessed, there was no statistically significant difference between the R group and RO group. This may be due to the fact that early histological improvement is not reflected in biomechanical strengths. This is because fibrovascular tissue formed during tendon-to-bone healing needs to advance into the bone and biomechanical endurance increases as this progress occurs [29]. As a result, it is seen that this healing process is not accelerated sufficiently with ozone alone.

The positive effects of PRP attributed to rotator cuff healing have been suggested to result from growth factors secreted from platelet granules [36]. PRP needs to be activated to increase the release of growth factors. Activation of platelets in PRP can be achieved by contacting with calcium chloride, thrombin, or collagen [29]. Cavallo et al. used CaCl_2_, autologous thrombin, CaCl_2_ + thrombin, and collagen type I as PRP activators in their study comparing PRP activating agents and evaluated VEGF, TGF-훽1, PDGF-AB, IL-1훽, and TNF-훼 releases. They found that type I caused the least GF release and CaCl_2_ showed a progressively increasing release from 15 min to 24 h [37].

Ozone therapy, which has been used in musculoskeletal diseases in recent years, can be used in PRP activation. PRP exposure to O_2_O_3_ has been shown to stimulate the release of different growth factors [38, 39]. Re et al. [18] found that the release of a number of growth factors, including PDGF, TGF-β1, and interleukin-8 (IL-8) in ozone-activated PRP, but not a significant increase in fibroblast growth factor (FGF) release. Bocci et al. [40] showed that IL-8, PDGF, and TGF-β increased with ozonation of PRP [40]. Platelet activation increases access to autologous growth factors that accelerate normal tissue regeneration processes. Similarly, in the early stages of wound healing, platelets are activated by thrombin and collagen, and growth factors are released from activated platelets to facilitate repair and healing [18].

The sequence of events leading to bone formation and tissue healing (chemotaxis, cell migration, proliferation, and differentiation) is regulated by growth factors mostly found in PRP [18]. In this study, we found better tendon-to-bone healing in the RPO group both histologically and biomechanically. This result in the RPO group may be due to the activation of PRP with O_2_O_3_ and the resulting growth factors in the repair region that lead to recovery. The development of the bone-tendon junction occurs primarily by chondrogenesis and tendonogenesis, followed by mineralization. However, fibrovascular tissue formed between the tendon and bone has to advance into the bone in order to be able to talk about a complete recovery [29]. The recovery after surgical repair is biomechanically weaker than normal rotator cuff. As a matter of fact, even in the RPO group in which we obtained the best results in our study, tensile strengths were approximately 77% of the values obtained from the intact supraspinatus tendon.

Our study has some limitations. First, as this is an animal study, the results may not always be consistent with clinical trials. Therefore, the results of this study should be supported by clinical studies. The second limitation is the application of PRP in the form of injection directly to the repair site. Losing some amount of injected PRP during the administration is inevitable and it is difficult to achieve a standard concentration at the repair site. It may be beneficial to use PRP embedding tissue-engineering implants to provide a standard dose in the repair area [5]. Our third limitation is the duration of our study, we ended our study at 6 weeks, but as is known, recovery after rotator cuff repair continues after 6 weeks. It is also necessary to investigate how the healing in the groups is affected over a longer period of time. In particular, biomechanical endurance increases over time, even after histological recovery. Furthermore, there are factors such as tendon retraction and osteoarthritis in the rotator cuff tears that are caused by long-term intrinsic degenerative changes. This situation cannot be simulated with an acute-induced and immediately repaired animal experiment. This situation emerges as another limitation to our study. Finally, in this study, we observed encouraging results, especially in the RPO group, and we thought that this was related to growth factors released by the activation of PRP with O_2_O_3_; however, immunohistochemical analyses testing the amounts and effects of these growth factors were not performed in this study. This is another limitation of our study.

In conclusion, our results indicate that ozonized PRP can be used as biological support to increase the rate of healing of rotator cuff repair. However, due to the limitations of this animal study, the results need to be supported by clinical studies. The advantages of PRP for tendon-to-bone healing and the ability of ozone to stimulate the release of growth factors are important milestones for future research in this area. In particular, studies to optimize the release of growth factors can provide significant progress in tendon-to-bone healing.

## Data Availability

The datasets used and/or analyzed during the current study are available from the corresponding author on reasonable request.

## Abbreviations

PRP: Platelet-rich plasma
R: Repair
RP: Repair + PRP
RO: Repair + ozone
RPO: Repair + PRP + ozone
BCC: Blood cell component
SEC: Serum component
PPP: Platelet poor plasma
TGF-β: Transforming growth factor-β
bFGF: Basic fibroblast growth factor
PDGF: Platelet-derived growth factor
VEGF: Vascular endothelial growth factor
CTGF: Connective tissue growth factor
EGF: Epidermal growth factor
IL-8: Interleukin-8
FGF: Fibroblast growth factor
